# Pre-Diagnosis Sleep Status and Survival after a Diagnosis of Ovarian Cancer: A Prospective Cohort Study

**DOI:** 10.3390/jcm11236914

**Published:** 2022-11-23

**Authors:** Xiaoying Li, Chang Gao, Yifan Wei, Zhaoyan Wen, Xinyu Li, Fanghua Liu, Tingting Gong, Shi Yan, Xue Qin, Song Gao, Yuhong Zhao, Qijun Wu

**Affiliations:** 1 Department of Clinical Epidemiology, Shengjing Hospital of China Medical University, Shenyang 110004, China; 2 Clinical Research Center, Shengjing Hospital of China Medical University, Shenyang 110004, China; 3 Liaoning Key Laboratory of Precision Medical Research on Major Chronic Disease, Shenyang 110004, China; 4 Department of Obstetrics and Gynecology, Shengjing Hospital of China Medical University, Shenyang 110004, China

**Keywords:** cohort, ovarian cancer, sleep, survival

## Abstract

**Objective:** To explore if pre-diagnosis sleep status is associated with overall survival (OS) of ovarian cancer (OC). **Methods:** This is a prospective cohort study of 853 OC patients newly diagnosed between 2015 and 2020. Sleep status was measured by the Pittsburgh Sleep Quality Index (PSQI). Vital status of patients was obtained through active follow-up and linkage to medical records and cancer registry. The Cox proportional hazards regression model was utilized to calculate hazard ratios (HRs) and 95% confidence intervals (CIs) for aforementioned associations. **Results:** During the follow-up period (median: 37.57 months, interquartile: 25.00 to 50.17 months), 123 (18.39%) OC patients died. The HR (95%CI) for OS of OC was 2.13 (1.42–3.18) for sleeping after 22:00, compared with sleeping before 22:00; 2.43 (1.64–3.62) for poor sleep quality, compared to good sleep quality; 2.26 (1.37–3.72) for late bed-early rise and 1.93 (1.09–3.42) for late bed-late rise, compared with early bed-early rise; 0.40 (0.24–0.67) for night sleep duration of ≥7.5 h/day, compared with 7–7.5 h/day; 0.53 (0.29–0.98) for total sleep duration of ≥8 h/day, compared with 7.5–8 h/day. Further, the interaction effects were significant between residual lesions and wake-up time, night bedtime, sleep pattern, and between total sleep duration and menopausal status, parity. Additionally, there was a significant curvilinear association between PSQI score and OS (*p* nonlinear <0.05). **Conclusions:** Pre-diagnosis longer total and night sleep duration were associated with better OS, whereas later sleeping time, poor sleep quality, and bad sleep patterns were associated with poor OS among OC survivors.

## 1. Introduction

Ovarian cancer (OC) is one of the most common gynecologic cancers with the highest mortality rate among women around the world [1,2,3]. According to the latest data from the International Agency for Research on Cancer, there were about 310,000 new cases of OC and 210,000 deaths worldwide in 2020 [2]. Due to a lack of screening tools and the non-specific symptoms, most OC cases are diagnosed at advanced disease stage [4]. In recent years, survival of OC has been slowly improving over time; however, the 5-year survival rate remains poor at less than 50% in China [5]. Previous studies suggested that OC women with similar cancer characteristics and similar treatments might have different outcomes [6,7]. That indicates that except for non-modifiable cancer characteristics (e.g., clinical stage, residual tumor size, and level of neoplastic differentiation) [8,9,10], modifiable factors may be involved in survival of OC. Therefore, identifying modifiable lifestyle factors related to survival of OC is worthy of attention.

Previous studies have found that circadian regulatory functions play critical roles in several hallmarks of cancer, including cell proliferation, cell death, DNA repair, and metabolic alteration [11,12,13]. These biological mechanisms suggest that sleep may be involved in the etiology and development of cancer. So far, although epidemiological studies have provided evidence of the associations between sleep and risk of diverse cancers including OC [14,15], few studies have explored the relationship between sleep and cancer survival. A report from the Women’s Health Initiative suggested that short pre-diagnosis sleep duration and frequent snoring were associated with worse breast cancer survival [16]. Moreover, a study from the UK Biobank indicated that a healthy sleep pattern was associated with the lower risk of cancer mortality [17]. However, to date, associations of sleep status with survival of OC have not been investigated.

Since the current lack of epidemiological evidence regarding the role of sleep status in OC survival, herein, we aim to first evaluate the associations between pre-diagnosis sleep status (e.g., sleep time, quality, and patterns) and overall survival (OS) among OC patients on the basis of a prospective cohort study-the OC follow-up study (OOPS)-with 853 newly diagnosed OC patients in China.

## 2. Materials and Methods

### 2.1. Study Population

**Figure 1:**
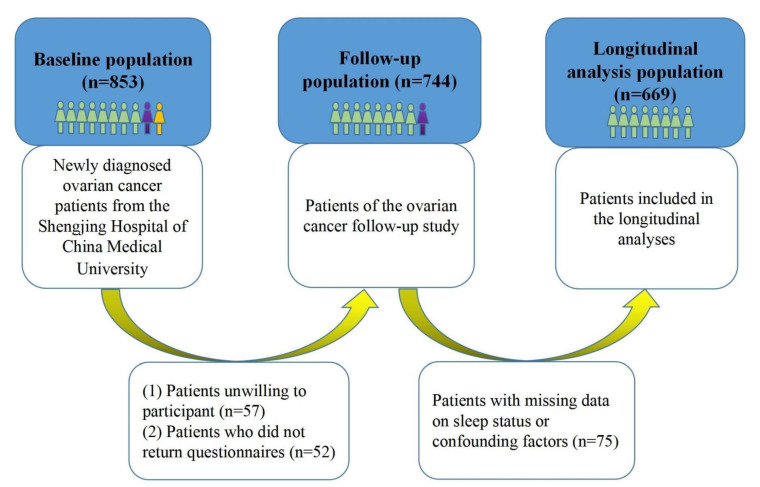
Participant flow chart.

The OOPS is a prospective cohort study of OC women, aiming to evaluate associations between demographic, clinical, and lifestyle factors and OC prognosis [18,19]. This study has been conducted at the gynecological oncology wards at Shengjing Hospital of China Medical University, Shenyang, China, since 2015. The patients should meet the following criteria: (1) women with newly diagnosed OC; (2) women with age at diagnosis between 18 and 79 years old; (3) women who are conscious and able to answer questionnaires. A total of 853 women with newly diagnosed OC were identified from January 2015 to December 2020. Among them, a total of 796 women (93.32%) agreed to participate. Of the 796 women, 744 (87.22%) women returned the study questionnaires. Additionally, we further excluded patients with missing data on sleep status or potential confounding factors (*n* = 75), leaving 669 eligible OC patients for the final analysis (Figure 1). The study was approved by the Institutional Review Board of the Ethics Committee of Shengjing Hospital of China Medical University and all the patients have signed the informed consent.

### 2.2. Data Collection

At baseline, we obtained data about individual characteristics and lifestyle factors from a self-administered questionnaire, which included information on smoking status, alcohol drinking status, tea drinking status, physical activity, electronic product use, education level, family income per month, menopausal status, parity, rotating night shift work, and sleep parameters. Clinical characteristics, including age at diagnosis, histological type, International Federation of Gynecology and Obstetrics (FIGO) stage, histopathologic grade, residual lesions, and comorbidities, were collected according to the electronic medical records of the Shengjing hospital information system. In addition, all women underwent a physical examination to measure height and weight by a trained staff following a standard protocol. Body mass index (BMI) was subsequently estimated by dividing weight in kilograms by height in squared meters. 

### 2.3. Exposure Assessment

The Pittsburgh sleep quality index (PSQI) questionnaire, a brief, reliable, valid, and standardized measure of sleep quality, consists of 19 items that reflects on seven major components of sleep: subjective sleep quality, sleep latency, sleep duration, sleep efficiency, sleep disturbance, use of sleeping medication, and daytime dysfunction due to sleepiness [20]. Women were asked to report their sleep characteristics during the month before OC diagnosis. Each component weights 0 to 3 scales equally. Thus, the total score of PSQI ranges from 0–21 points and the score higher than five indicates poor quality of sleep [20]. This method has been previously used in studies of different health outcomes [21,22,23]. In addition, patients were asked if they had a midday nap habit during the month before OC diagnosis. If the answer was affirmative, they were further asked for the usual midday nap duration. Total sleep duration corresponds to the sum of daytime napping and night sleep duration. 

### 2.4. Follow-Up and Outcome

The outcome of this study was death due to any cause. The vital status of patients was obtained through active follow-up as well as linkage to medical records and cancer registry every six months. Survival time was defined as the time from OC diagnosis to all-cause death, or the date of last follow-up (31 March 2021) for patients who were still alive.

### 2.5. Statistical Analysis

Attributes of the study population were compared across sleep quality categories, using Student’s t-tests for continuous variables, and the Chi-square tests for categorical variables. Results were presented as mean  ±  standard deviation for continuous variables, and counts with percentages for categorical variables. We used the Kaplan–Meier technique to estimate crude OS probabilities and plot crude survival curves. The Cox proportional hazards regression model was utilized to estimate the hazard ratios (HRs) and two-sided 95% confidence intervals (CIs) for the associations of pre-diagnosis sleep status with OS. Firstly, a crude Cox regression model without any adjustment was applied, then two adjusted models were performed. Model 1 was adjusted for age at diagnosis and BMI, while model 2 was additionally adjusted for physical activity, electronic product use, smoking status, alcohol drinking status, tea drinking status, education level, family income per month, menopausal status, parity, histological type, histopathologic grade, FIGO stage, residual lesions, comorbidities, and rotating night shift work based on model 1. Before the Cox proportional hazards regression model, we examined the proportional hazards assumption and no violations were observed (all *p* > 0.05).

In our analyses, pre-diagnosis sleep parameters included night bedtime, wake-up time, night sleep duration, daytime napping duration, total sleep duration, sleep quality, and sleep pattern. In a previous study on sleep and risk of OC, night sleep duration of 7 h/day was taken as the reference [15]. Therefore, with a 30 min interval, we used sleep duration of 7–7.5 h per night as a reference. Further, 7.5–8 h/day was taken as a reference for total sleep duration. In addition, a study on night bedtime and health among middle-aged and older adults took “before 10:00” as the reference [24]. Since OC patients are mostly middle-aged and elderly, we also adopted “before 10:00” as a reference for night bedtime in our study. Correspondingly, “before 6:00” was used as a reference for wake-up time. Further, sleep patterns were divided into four groups based on night bedtime and wake-up time: early bed-early rise (before 22:00 and before 6:00, reference group), early bed-late rise (before 22:00 and after 6:00), late bed-early rise (after 22:00 and before 6:00), and late bed-late rise (after 22:00 and after 6:00).

Stratified analyses were further carried out according to age at diagnosis, BMI, menopausal status, parity, histological type, and residual lesions. Multiplicative model interaction effects of the sleep parameters with these stratifying variables were evaluated by adding the cross-product terms to the multivariable-adjusted Cox regression model, respectively. Additive model interaction effects were assessed based on Tomas Andersson’s study and did not exist if the 95%CIs of the relative excess risk due to interaction (RERI) or the attributable proportion (AP) contained 0, or 95%CIs of the synergy index (S) contained 1 [25]. Non-linear associations between sleep status and OS of OC were explored using restricted cubic spline [26]. *p* < 0.05 (two-sided) was considered statistically significant and all analyses were carried out using SAS version 9.4 (SAS Institute, Cary, NC, USA).

## 3. Results

General characteristics of OC patients stratified by sleep quality were shown in Table 1. Compared to patients with good sleep quality, those with poor sleep quality had longer time for electronic products using, shorter total and night sleep duration, and were more likely to be alcohol drinkers. During the follow-up period (median: 37.57 months, interquartile: 25.00 to 50.17 months), 18.39% (*n* = 123) of the patients died. Non-serous histological type, higher FIGO stage, and larger residual lesions lowered OS of OC (Table 2).

**Figure 2:**
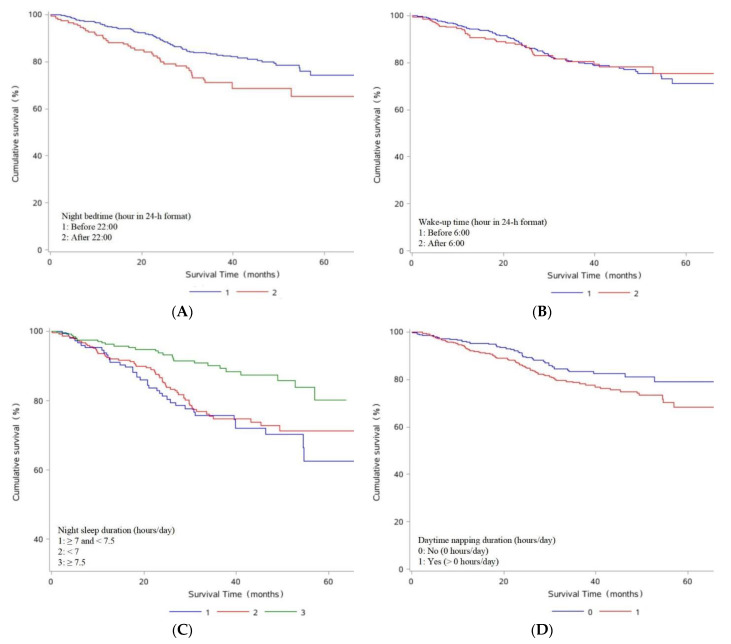

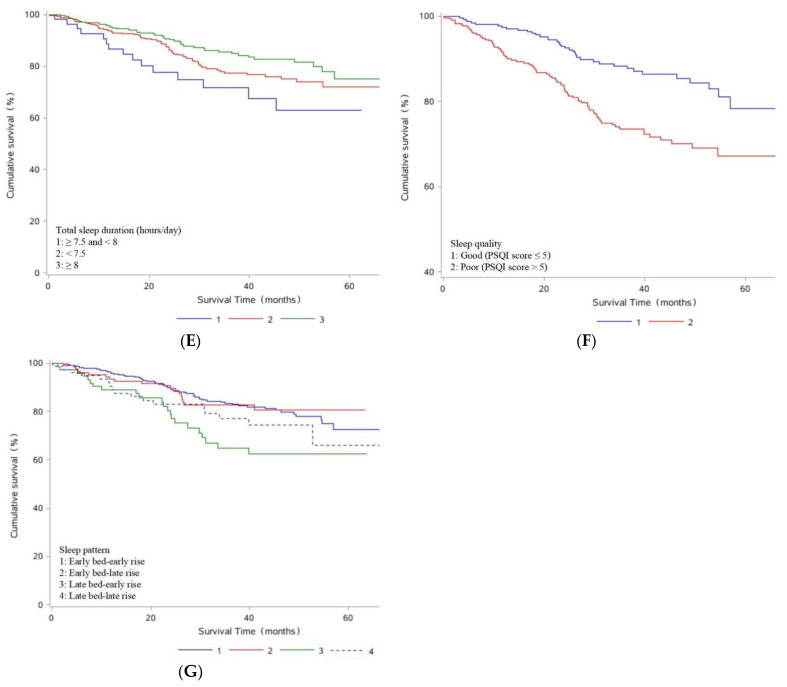
Kaplan–Meier survival curves for night bedtime (**A**), wake-up time (**B**), night sleep duration (**C**), daytime napping duration (**D**), total sleep duration (**E**), sleep quality (**F**), and sleep pattern (**G**).

Associations between pre-diagnosis sleep status and OS among OC patients were summarized in Table 3 and Figure 2. Patients who slept after 22:00 had worse OS compared to those who slept before 22:00 (HR: 2.13, 95% CI: 1.42–3.18). Additionally, going to bed every 30 min later was associated with worse OS (HR: 1.49, 95% CI: 1.03–2.16). Compared with a night sleep duration of 7–7.5 h/day, longer night sleep duration improved OS (HR: 0.40, 95% CI: 0.24–0.67). Similarly, longer total sleep duration (≥8 h/day) improved OS compared to a total sleep duration of 7.5–8 h/day (HR: 0.53, 95%CI: 0.29–0.98). Additionally, poor sleep quality was significantly associated with worse OS compared to good sleep quality (HR: 2.43, 95% CI: 1.64–3.62). Notably, every one-point increase in the PSQI was related to worse OS (HR: 1.08, 95% CI: 1.04–1.13). Moreover, compared with OC patients who went to bed and got up early, those with sleep patterns of late bed-early rise (HR: 2.26, 95% CI: 1.37–3.72) or late bed-late rise (HR: 1.93, 95% CI: 1.09–3.42) had worse OS.

We further carried out stratified analyses and interaction effect analyses based on relevant characteristics (Tables S1–S6). Interestingly, there were significant multiplicative model interaction effects between wake-up time and residual lesions (*p =* 0.01), total sleep duration and menopausal status (*p =* 0.01) and parity (*p =* 0.02), sleep pattern and residual lesions (*p =* 0.02) on OS (Tables S1, S2 and S5). In addition, additive model interaction effects between night bedtime and residual lesions were statistically significant (RERI: 3.22, 95% CI: 0.07–6.38; AP: 0.58, 95% CI: 0.30–0.86; S: 3.43, 95% CI: 1.26–9.37) (Table S6). Correspondingly, associations between these sleep parameters and OS of OC only existed in certain subgroups. For example, longer total sleep duration was associated with OS of OC only in postmenopausal women (≥8 h/day vs. 7.5–8 h/day: HR = 0.38, 95% CI: 0.19–0.77), while shorter total sleep duration was related to OS of OC only in non-menopausal women (<7.5 h/day vs. 7.5–8 h/day: HR = 6.91, 95% CI: 1.32–36.29) (Table S2).

**Figure 3:**
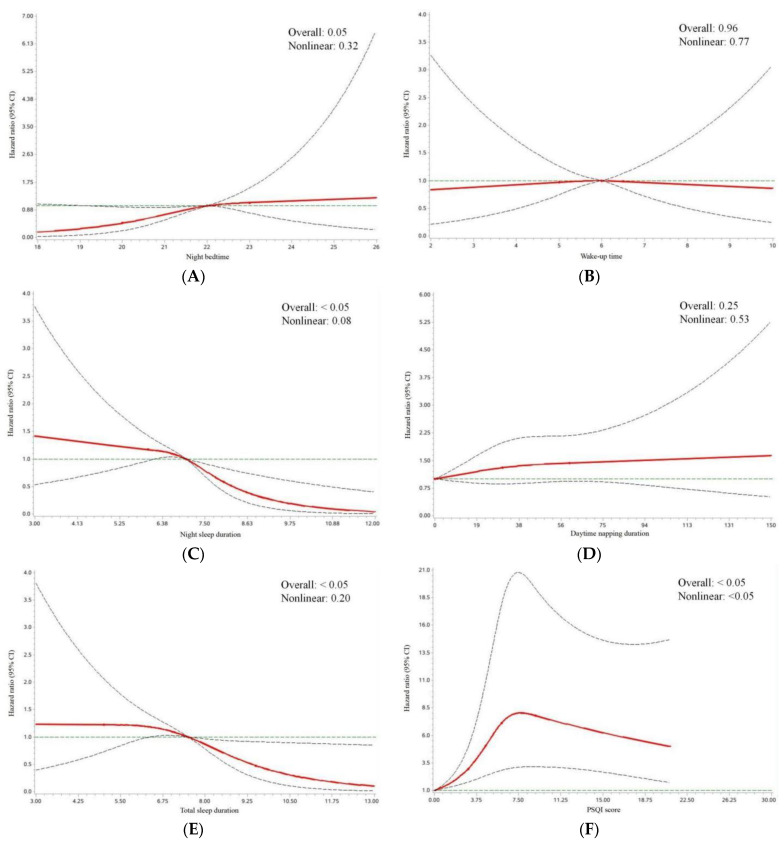
HR and 95% CI of overall survival among ovarian cancer patients by (**A**) night bedtime; (**B**) wake-up time; (**C**) night sleep duration; (**D**) daytime napping duration; (**E**) total sleep duration; (**F**) PSQI score. Multivariable HRs and 95% CIs for overall survival among ovarian cancer patients across strata of various factors. Cox model was adjusted for age at diagnosis, body mass index, physical activity, electronic product use, smoking, alcohol drinking, tea drinking, education level, family income per month, menopausal status, parity, histological type, histopathologic grade, FIGO stage, residual lesions, comorbidities, and rotating night shift work. The red line and dashed line represented the estimated HRs and their 95% CIs, respectively. CI, confidence interval; HR, hazard ratio; PSQI, Pittsburgh sleep quality index.

We further explored the non-linear associations between pre-diagnosis sleep status and OS (Figure 3) and found a curvilinear association between PSQI score and OS (*p*_nonlinear_ <0.05). OC patients had the worst OS with a PSQI score of 7.7 to 7.8 (HR = 8.05).

## 4. Discussion

In this prospective cohort study, we observed that sleeping after 22:00, poor sleep quality, and bad sleep patterns (late bed-early rise and late bed-late rise) were associated with worse OS, whereas longer total and night sleep duration were related to improved OS among OC patients. Noteworthy, the interaction effects were significant for residual lesions and wake-up time, night bedtime, sleep pattern, and for total sleep duration and menopausal status, parity. In addition, we found a curvilinear association between PSQI score and OS. 

So far, there have been no studies investigating the association between pre-diagnosis sleep status and OC prognosis. Only two observational studies investigated the relationship between sleep and risk of OC [14,15]. A prospective cohort study, comprised of 101,609 adult females participating in the California Teachers Study, found that increasing sleep duration was associated with the increased risk of estrogen-mediated cancers (*p* = 0.04) [14]. Besides, Liang et al., found restful or very restful sleep quality was associated with a lower risk of invasive serous OC (HR: 0.73, 95% CI: 0.60–0.90) based on the Women’s Health Initiative [15]. Moreover, several previous studies indicated that pre-diagnosis sleep status might modify the progression of certain cancers such as breast cancer, lung cancer, and colorectal cancer [16,17,27,28,29]. Phipps et al., found pre-diagnosis short sleep duration combined with frequent snoring might influence subsequent breast cancer survival based on the Women’s Health Initiative [16]. Gottfried et al., observed self-reported sleep quality might be a prognostic indicator for lung cancer survival in a cohort of 404 lung cancer patients [27].

Potential biological mechanisms linking sleep and cancer may include the following pathways. At first, sleep duration and quality can affect circadian rhythm. Escape from circadian regulation may enable the transformation of normal cells to malignant cells by some mechanisms, including sustaining proliferative signal, enabling replicative immortality, deregulating cellular metabolism, and so on; carcinogenesis, in turn, inhibits homeostasis balance imposed by the circadian clock, further promoting the occurrence and development of cancer [13,30,31,32]. Secondly, short sleep duration may reduce melatonin release [33,34]. Melatonin is mainly synthesized by the pineal gland during the night and associated with a variety of physiological functions, including anticancer effects [35,36]. In addition, the biological mechanisms between sleep and cancer may be related to inflammation and cytokines. A prospective study of 294 gastrointestinal cancer patients found that sleep duration was associated with symptom burden and poorer survival and IL-2 might mediate the association between sleep and survival [37].

Our study found several interaction effects between sleep status and potential confounders on OS among OC patients. Worse survival was noted among OC patients with waking up after 6:00, late bed-early rise, or late bed-late rise only in subgroups with residual lesions. It is possible that OC patients with residual lesions have worse physical status and prognosis compared to those without residual lesions [9], and as a result, they are more susceptible to sleep status. Nevertheless, although these interaction effects were observed, we could not rule out the possibility of chance findings for some reasons, for example, the relatively small sample sizes of some subgroups. Future studies with larger sample size are warranted to provide further insight into the aforementioned effects. Additionally, this study identified a curvilinear association between PSQI score and OS of OC with a peak HR equal to 8.05. That meant OS of OC decreased with the increase of the PSQI score when PSQI score was less than 7.7, improved as the PSQI score increased when the PSQI score was greater than 7.8, and was worst with a PSQI score of 7.7 to 7.8. A plausible explanation for this phenomenon might be that poor sleep quality with a PSQI score of more than 7.8 is more likely to significantly affect people’s quality of life, and therefore more likely to be treated after the OC diagnosis, than with a PSQI score of less than 7.7. However, the exact mechanism is subject to further study.

Compared to OC patients with good sleep quality, those with poor sleep quality were more likely to be alcohol drinkers in this study. A plausible explanation for this phenomenon might be that pain is related to poor sleep among cancer patients, and alcohol drinking is one of the ways that people relieve pain [38,39]. In addition, our study found that poor sleep quality was more strongly associated with worse OS of OC among patients who drank alcohol (HR: 1.34, 95% CI: 1.16–1.54) than among non-drinkers (HR: 1.06, 95% CI: 1.01–1.12). Therefore, alcohol drinking was adjusted for as a potential confounding factor in this study.

Several crucial strengths of this study deserve mention. First, to our best knowledge, this is the first study exploring associations between pre-diagnosis sleep status and OS among OC patients. Our findings might be applied to improve cancer management. For example, people at high risk of OC should pay particular attention to getting enough sleep, good sleep quality, and avoiding staying up late. Additionally, when sleep appears abnormal, it should be actively intervened. Previous studies have shown that sleep can be improved before, during, and after cancer treatment through certain interventions, such as cognitive behavioral therapy, a regular sleep schedule, limit caffeine intake, exercise therapy, ambient noise limitation, melatonin and other medications [40]. Second, our investigation had a prospective design, characterized by high baseline survey participation rate and follow-up retention rate, which could reduce the potential for recall bias and selection bias. Third, a diverse array of potential confounders were rigorously adjusted in our analyses, such as electronic product use, comorbidities, FIGO stage, histological type, and residual lesions, which enhanced the reliability of findings. Fourth, we explored multiplicative and additive model interaction effects between sleep status and several potential confounders, and found significant results. In addition, we found a curvilinear association between PSQI score and OS of OC.

Several limitations should be taken into account when interpreting our findings. First, sleep status was collected through self-report, which might induce misclassification, recall bias or social desirability answers. Additionally, this study only focused on the sleep status of the one month before diagnosis, which might not reflect the long-term sleep status of OC patients, resulting in reduced reliability of our findings. However, we selected well-trained investigators to collect information on sleep status among OC patients and PSQI has become the most common subjective measurement method of sleep problems, which might minimize deviation. Second, since our current study only collected data on pre-diagnosis sleep status in OC patients, it did not represent post-diagnosis sleep status. Additionally, researches on the differences between pre-diagnosis sleep and post-diagnosis sleep and the effect of post-diagnosis sleep on the survival of OC patients are scarce. Thus, future studies are necessary to further explore the aforementioned associations. Third, associations between pre-diagnosis sleep status and progression-free survival (PFS) among OC patients were not explored in our investigation. However, PFS might be similar to OS among OC patients because of the high mortality rate. Fourth, a common concern of any observational study is residual confounding factors. In the same way, although we comprehensively adjusted for the majority of potential confounders to minimize their impact, we failed to exclude the possibility of unknown or unmeasured residual confounders. For example, pre-diagnosis pain sate of OC patients may affect sleep and therefore is a potential confounder for associations between pre-diagnosis sleep status and OS of OC [41]. However, data on pre-diagnosis pain control among OC patients were unavailable in this study and therefore failed to be adjusted. In the near future, we will investigate associations of post-diagnosis sleep status with OS of OC and adjust for post-diagnosis pain control. Finally, populations from different regions may have different sleep habits. Thus, this study’s findings should be extrapolated with caution to other countries and regions.

## 5. Conclusions

Our findings revealed that pre-diagnosis night sleep duration over 7.5 h/day and total sleep duration over 8 h/day improved OS, whereas sleeping after 22:00, poor sleep quality and bad sleep patterns (late bed-early rise and late bed-late rise) lowered OS among OC patients. Further studies with longer follow-up periods are warranted to confirm our findings.

## Figures and Tables

**Table 1:** General characteristics of ovarian cancer patients according to sleep quality (N = 669). Values are numbers (percentages) unless stating otherwise.

Variables	Sleep Quality		*p* Value
	Good (PSQI Score ≤ 5)	Poor (PSQI Score > 5)	
No. of patients	317	352	
Mean (SD) age at diagnosis (years)	53.61 ± 9.60	53.78 ± 9.72	0.82
Mean (SD) body mass index (kg/m^2^)	23.04 ± 3.54	23.41 ± 3.64	0.19
Mean (SD) physical activity (MET/hours/week)	112.09 ± 79.16	108.33 ± 79.43	0.54
Mean (SD) electronic product use ^a^ (hours/week)	20.99 ± 12.31	23.05 ± 14.62	<0.05
Educational level			0.57
Junior secondary or below	168 (53.00)	186 (52.84)	
Senior high school/technical secondary school	63 (19.87)	80 (22.73)	
Junior college/university or above	86 (27.13)	86 (24.43)	
Family income per month (Yuan)			0.81
<5000	194 (61.20)	208 (59.09)	
5000 to <10,000	87 (27.44)	99 (28.13)	
≥10,000	36 (11.36)	45 (12.78)	
Parity			0.22
≤1	236 (74.45)	247 (70.17)	
≥2	81 (25.55)	105 (29.83)	
Menopausal status (yes)	220 (69.40)	259 (73.58)	0.23
Cigarette smoking (yes)	27 (8.52)	38 (10.80)	0.32
Alcohol drinking (yes)	52 (16.40)	87 (24.72)	<0.05
Tea drinking (yes)	108 (34.07)	107 (30.40)	0.31
Rotating night shift work (yes)	21 (6.62)	33 (9.38)	0.19
Taking a nap during the day (yes)	184 (58.04)	227 (64.49)	0.09
Mean (SD) night bedtime (hour in 24 h format)	21.49 ± 1.43	21.62 ± 2.12	0.37
Mean (SD) wake-up time (hour in 24 h format)	5.97 ± 0.92	5.88 ± 1.29	0.26
Mean (SD) night sleep duration (hours/day)	7.67 ± 0.86	6.11 ± 1.14	<0.05
Mean (SD) daytime napping duration ^b^ (minutes/day)	44.48 ± 23.66	45.24 ± 24.27	0.75
Mean (SD) total sleep duration (hours/day)	8.10 ± 0.99	6.59 ± 1.29	<0.05

MET, metabolic equivalent; PSQI, Pittsburgh sleep quality index; SD, standard deviation. ^a^ Electronic products included television, computer, mobile phone, iPad, and so on. ^b^ Daytime napping duration among participants who took a nap during the day.

**Table 2:** Associations between clinical characteristics and all-cause mortality among women diagnosed with ovarian cancer.

Characteristics	No. of Deaths/Total (%)	Adjusted HR ^a^ (95% CI)
Age at diagnosis		
≤50	43/246 (17.48)	1.00 (ref)
>50	80/423 (18.91)	1.19 (0.82, 1.74)
Histological type		
Serous	88/458 (19.21)	1.00 (ref)
Non-serous	35/211 (16.59)	1.69 (1.07, 2.66)
Histopathologic grade		
Poorly differentiated	112/569 (19.68)	1.00 (ref)
Moderately differentiated	7/48 (14.58)	0.66 (0.30, 1.48)
Well differentiated	4/52 (7.69)	0.49 (0.18, 1.36)
FIGO stage		
Ⅰ–Ⅱ	39/335 (11.64)	1.00 (ref)
Ⅲ–Ⅳ	84/312 (26.92)	2.73 (1.75, 4.26)
Residual lesions		
No	77/523 (14.72)	1.00 (ref)
≤1 cm	30/104 (28.85)	1.66 (1.06, 2.60)
>1 cm	16/42 (38.10)	2.33 (1.32, 4.09)
Comorbidities		
No	69/367 (18.80)	1.00 (ref)
Yes	54/302 (17.88)	0.97 (0.67, 1.39)

CI, confidence interval; FIGO, The International Federation of Gynecology and Obstetrics; HR, hazard ratio; Ref, reference. HR and 95% CI were calculated with the use of the Cox proportional hazards regression model. ^a^ Mutually adjusted for all other variables listed in the table.

**Table 3:** Associations between pre-diagnosis sleep status and overall survival among ovarian cancer patients.

Sleep Parameters	Deaths, N (% of Total Deaths)	Model 1 ^a^ HR (95% CI)	Model 2 ^b^ HR (95% CI)
Night bedtime (hour in 24 h format)			
Before 22:00	83/518 (16.02)	1.00 (Ref)	1.00 (Ref)
After 22:00	40/151 (26.49)	1.73 (1.19, 2.53)	2.13 (1.42, 3.18)
the change of every 30 min		1.39 (0.99, 1.95)	1.49 (1.03, 2.16)
Wake-up time (hour in 24 h format)			
Before 6:00	86/465 (18.49)	1.00 (Ref)	1.00 (Ref)
After 6:00	37/204 (18.14)	1.03 (0.70, 1.52)	1.07 (0.71, 1.61)
the change of every 30 min		0.95 (0.69, 1.32)	1.00 (0.71, 1.41)
Night sleep duration (hours/day)			
<7	58/276 (21.01)	0.92 (0.61, 1.39)	0.90 (0.59, 1.37)
≥7 and <7.5	39/148 (26.35)	1.00 (Ref)	1.00 (Ref)
≥7.5	26/245 (10.61)	0.43 (0.26, 0.71)	0.40 (0.24, 0.67)
Daytime napping duration (hours/day)			
No (0 h/day)	38/258 (14.73)	1.00 (Ref)	1.00 (Ref)
Yes (> 0 h/day)	85/411 (20.68)	1.49 (1.01, 2.18)	1.37 (0.93, 2.03)
the change of every 30 min		1.20 (1.02, 1.41)	1.15 (0.97, 1.37)
Total sleep duration (hours/day)			
<7.5	65/330 (19.70)	0.61 (0.35, 1.08)	0.80 (0.44, 1.47)
≥7.5 and <8	15/55 (27.27)	1.00 (Ref)	1.00 (Ref)
≥8	43/284 (15.14)	0.43 (0.24, 0.78)	0.53 (0.29, 0.98)
Sleep quality			
Good (PSQI score ≤ 5)	39/317 (12.30)	1.00 (Ref)	1.00 (Ref)
Poor (PSQI score > 5)	84/352 (23.86)	2.31 (1.57, 3.39)	2.43 (1.64, 3.62)
the change of every 1 score		1.08 (1.04, 1.13)	1.08 (1.04, 1.13)
Sleep pattern			
Early bed-early rise	64/392 (16.33)	1.00 (Ref)	1.00 (Ref)
Early bed-late rise	19/126 (15.08)	1.00 (0.60, 1.68)	0.97 (0.57, 1.64)
Late bed-early rise	22/73 (30.14)	2.05 (1.26, 3.32)	2.26 (1.37, 3.72)
Late bed-late rise	18/78 (23.08)	1.46 (0.86, 2.47)	1.93 (1.09, 3.42)

CI, confidence interval; HR, hazard ratio; PSQI, Pittsburgh sleep quality index; Ref, reference. HR and 95% CI were calculated with the use of the Cox proportional hazards regression model. ^a^ Model 1: adjusted for age at diagnosis and body mass index. ^b^ Model 2: same as model 1 and further adjusted for physical activity, electronic product use, smoking, alcohol drinking, tea drinking, education level, family income per month, menopausal status, parity, histological type, histopathologic grade, FIGO stage, residual lesions, comorbidities, and rotating night shift work.

## Data Availability

Data are available upon request from the corresponding author.

## Supplementary Materials

The following supporting information can be downloaded at: https://www.mdpi.com/article/10.3390/jcm11236914/s1, Table S1: Subgroup analyses of the associations between night bedtime, wake-up time and overall survival among ovarian cancer patients. Table S2: Subgroup analyses of the associations between night sleep duration, total sleep duration and overall survival among ovarian cancer patients. Table S3: Subgroup analyses of the associations between daytime napping duration and overall survival among ovarian cancer patients. Table S4: Subgroup analyses of the associations between sleep quality and overall survival among ovarian cancer patients. Table S5: Subgroup analyses of the associations between sleep pattern and overall survival among ovarian cancer patients. Table S6: Additive model interaction effects between sleep and confounding factors on overall survival among ovarian cancer patients.
