# Supplementary material for: Pre-Diagnosis Sleep Status and Survival after a Diagnosis of Ovarian Cancer: A Prospective Cohort Study
Source: J Clin Med. 2022 Nov 23;11(23):6914. doi: 10.3390/jcm11236914 (PMC9741188; doi:10.3390/jcm11236914)
Supplement: Supplementary file 1 [file jcm-11-06914-s001.zip › jcm-1994516-supplementary.pdf]

Table S1. Subgroup analyses of the associations between night bedtime, wake-up time and overall survival among ovarian cancer patients

Variables	Night bedtime	Model 2 [a] HR (95% CI)	*P* for multiplicative interaction	Wake-up time	Model 2 [a] HR (95% CI)	*P* for multiplicative interaction
**Age at diagnosis (years)**	**≤ 50**		0.16	**≤ 50**		0.40
	Before 22:00	1.00 (Ref)		Before 6:00	1.00 (Ref)	
	After 22:00	**3.37 (1.53, 7.41)**		After 6:00	0.67 (0.32, 1.37)	
	the change of every 30 minutes	1.89 (0.91, 3.92)		the change of every 30 minutes	0.89 (0.46, 1.74)	
	**> 50**			**> 50**		
	Before 22:00	1.00 (Ref)		Before 6:00	1.00 (Ref)	
	After 22:00	**1.76 (1.04, 2.97)**		After 6:00	1.32 (0.79, 2.19)	
	the change of every 30 minutes	1.34 (0.84, 2.13)		the change of every 30 minutes	1.04 (0.69, 1.56)	
**Body mass index (kg/m^2)**	**< 24**		0.55	**< 24**		0.53
	Before 22:00	1.00 (Ref)		Before 6:00	1.00 (Ref)	
	After 22:00	**2.02 (1.14, 3.61)**		After 6:00	1.00 (0.58, 1.72)	
	the change of every 30 minutes	1.54 (0.95, 2.48)		the change of every 30 minutes	0.98 (0.64, 1.51)	
	**≥ 24**			**≥ 24**		

	Before 22:00	1.00 (Ref)		Before 6:00	1.00 (Ref)	
	After 22:00	**2.58 (1.30, 5.11)**		After 6:00	1.08 (0.56, 2.08)	
	the change of every 30 minutes	1.35 (0.67, 2.71)		the change of every 30 minutes	0.94 (0.53, 1.69)	
	**No**			**No**		
	Before 22:00	1.00 (Ref)		Before 6:00	1.00 (Ref)	
	After 22:00	**2.40 (1.02, 5.67)**		After 6:00	0.59 (0.26, 1.33)	
**Menopausal status**	the change of every 30 minutes	1.24 (0.56, 2.77)	0.87	the change of every 30 minutes	0.58 (0.28, 1.20)	0.42
	**Yes**			**Yes**		
	Before 22:00	1.00 (Ref)		Before 6:00	1.00 (Ref)	
	After 22:00	**2.13 (1.30, 3.50)**		After 6:00	1.21 (0.74, 1.98)	
	the change of every 30 minutes	1.54 (0.98, 2.42)		the change of every 30 minutes	1.12 (0.75, 1.67)	
	**≤ 1**			**≤ 1**		
	Before 22:00	1.00 (Ref)		Before 6:00	1.00 (Ref)	
	After 22:00	**2.23 (1.36, 3.64)**		After 6:00	1.02 (0.63, 1.66)	0.85
**Parity**	the change of every 30 minutes	**1.57 (1.02, 2.40)**	0.66	the change of every 30 minutes	0.89 (0.59, 1.32)	
	**≥ 2**			**≥ 2**		
	Before 22:00	1.00 (Ref)		Before 6:00	1.00 (Ref)	

	After 22:00	**2.90 (1.15, 7.31)**		After 6:00	1.18 (0.52, 2.65)	
	the change of every 30 minutes	1.65 (0.73, 3.77)		the change of every 30 minutes	1.81 (0.75, 4.38)	
**Histological type**	**Serous**		0.28	**Serous**		0.67
	Before 22:00	1.00 (Ref)		Before 6:00	1.00 (Ref)	
	After 22:00	**2.35 (1.45, 3.80)**		After 6:00	1.13 (0.71, 1.82)	
	the change of every 30 minutes	**1.62 (1.06, 2.47)**		the change of every 30 minutes	1.06 (0.72, 1.58)	
	**Non-serous**			**Non-serous**		
	Before 22:00	1.00 (Ref)		Before 6:00	1.00 (Ref)	
	After 22:00	1.39 (0.60, 3.20)		After 6:00	0.77 (0.28, 2.10)	
	the change of every 30 minutes	0.84 (0.37, 1.91)		the change of every 30 minutes	0.79 (0.39, 1.61)	
**Residual lesions**	**No**		0.09	**No**		**0.01**
	Before 22:00	1.00 (Ref)		Before 6:00	1.00 (Ref)	
	After 22:00	1.60 (0.96, 2.68)		After 6:00	0.71 (0.42, 1.21)	
	the change of every 30 minutes	1.20 (0.73, 1.96)		the change of every 30 minutes	0.83 (0.54, 1.27)	
	**Yes**			**Yes**		
	Before 22:00	1.00 (Ref)		Before 6:00	1.00 (Ref)	
	After 22:00	**4.31 (2.00, 9.26)**		After 6:00	**2.33 (1.17, 4.65)**	
	the change of every 30 minutes	**2.27 (1.16, 4.44)**		the change of every 30 minutes	**1.95 (1.01, 3.75)**	

CI, confidence interval; HR, hazard ratio; Ref, reference.

HR and 95% CI were calculated with the use of the Cox proportional hazards regression model.

[a] Adjusted for age at diagnosis, body mass index, physical activity, electronic product use, smoking, alcohol drinking, tea drinking, education level, family income per month, menopausal status, parity, histological type, histopathologic grade, FIGO stage, residual lesions, comorbidities, and rotating night shift work, unless a certain covariable is the basis of the stratification.

Table S2. Subgroup analyses of the associations between night sleep duration, total sleep duration and overall survival among ovarian cancer patients

Variables	Night sleep duration	Model 2 [a] HR (95% CI)	*P* for multiplicative interaction	Total sleep duration	Model 2 [a] HR (95% CI)	*P* for multiplicative interaction
**Age at diagnosis (years)**	**≤ 50**		0.58	**≤ 50**		0.16
	< 7	1.17 (0.45, 2.75)		< 7.5	1.84 (0.61, 5.58)	
	≥ 7 and < 7.5	1.00 (Ref)		≥ 7.5 and < 8	1.00 (Ref)	
	≥ 7.5	0.40 (0.15, 1.08)		≥ 8	0.92 (0.30, 2.78)	
	**> 50**			**> 50**		
	< 7	0.77 (0.45, 1.30)		< 7.5	**0.46 (0.22, 0.96)**	
	≥ 7 and < 7.5	1.00 (Ref)		≥ 7.5 and < 8	1.00 (Ref)	
	≥ 7.5	**0.38 (0.20, 0.73)**		≥ 8	**0.35 (0.16, 0.74)**	
**Body mass index (kg/m²)**	**< 24**		0.63	**< 24**		0.18
	< 7	1.09 (0.61, 1.94)		< 7.5	0.61 (0.29, 1.25)	
	≥ 7 and < 7.5	1.00 (Ref)		≥ 7.5 and < 8	1.00 (Ref)	
	≥ 7.5	**0.39 (0.20, 0.76)**		≥ 8	**0.31 (0.15, 0.66)**	
	**≥ 24**			**≥ 24**		

	< 7	0.87 (0.42, 1.78)		< 7.5	1.76 (0.49, 6.27)	
	≥ 7 and < 7.5	1.00 (Ref)		≥ 7.5 and < 8	1.00 (Ref)	
	≥ 7.5	**0.39 (0.16, 0.94)**		≥ 8	1.40 (0.38, 5.08)	
**Menopausal status**	**No**		0.34	**No**		**0.01**
	< 7	1.27 (0.49, 3.32)		< 7.5	**6.91 (1.32, 36.29)**	
	≥ 7 and < 7.5	1.00 (Ref)		≥ 7.5 and < 8	1.00 (Ref)	
	≥ 7.5	**0.30 (0.10, 0.91)**		≥ 8	2.77 (0.54, 14.24)	
	**Yes**			**Yes**		
	< 7	0.86 (0.51, 1.45)		< 7.5	0.52 (0.26, 1.02)	
	≥ 7 and < 7.5	1.00 (Ref)		≥ 7.5 and < 8	1.00 (Ref)	
	≥ 7.5	**0.46 (0.25, 0.85)**		≥ 8	**0.38 (0.19, 0.77)**	
**Parity**	**≤ 1**		0.13	**≤ 1**		**0.02**
	< 7	1.08 (0.65, 1.81)		< 7.5	1.09 (0.51, 2.35)	
	≥ 7 and < 7.5	1.00 (Ref)		≥ 7.5 and < 8	1.00 (Ref)	
	≥ 7.5	**0.38 (0.19, 0.73)**		≥ 8	0.62 (0.28, 1.38)	
	**≥ 2**			**≥ 2**		
	< 7	0.48 (0.18, 1.26)		< 7.5	0.40 (0.13, 1.22)	

	≥ 7 and < 7.5	1.00 (Ref)		≥ 7.5 and < 8	1.00 (Ref)	
	≥ 7.5	0.40 (0.14, 1.13)		≥ 8	0.38 (0.12, 1.21)	
**Histological type**	**Serous**		0.94	**Serous**		0.67
	< 7	0.87 (0.52, 1.45)		< 7.5	0.57 (0.29, 1.14)	
	≥ 7 and < 7.5	1.00 (Ref)		≥ 7.5 and < 8	1.00 (Ref)	
	≥ 7.5	**0.39 (0.21, 0.71)**		≥ 8	**0.35 (0.17, 0.72)**	
	**Non-serous**			**Non-serous**		
	< 7	1.11 (0.47, 2.62)		< 7.5	2.17 (0.55, 8.53)	
	≥ 7 and < 7.5	1.00 (Ref)		≥ 7.5 and < 8	1.00 (Ref)	
	≥ 7.5	**0.31 (0.10, 0.96)**		≥ 8	1.29 (0.33, 5.13)	
**Residual lesions**	**No**		0.11	**No**		0.38
	< 7	1.30 (0.73, 2.30)		< 7.5	1.09 (0.46, 2.59)	
	≥ 7 and < 7.5	1.00 (Ref)		≥ 7.5 and < 8	1.00 (Ref)	
	≥ 7.5	**0.40 (0.20, 0.79)**		≥ 8	0.57 (0.23, 1.37)	
	**Yes**			**Yes**		
	< 7	0.56 (0.25, 1.26)		< 7.5	0.70 (0.27, 1.82)	
	≥ 7 and < 7.5	1.00 (Ref)		≥ 7.5 and < 8	1.00 (Ref)	
	≥ 7.5	0.52 (0.21, 1.25)		≥ 8	0.74 (0.28, 1.91)	

CI, confidence interval; HR, hazard ratio; Ref, reference.

HR and 95% CI were calculated with the use of the Cox proportional hazards regression model.

[a] Adjusted for age at diagnosis, body mass index, physical activity, electronic product use, smoking, alcohol drinking, tea drinking, education level, family income per month, menopausal status, parity, histological type, histopathologic grade, FIGO stage, residual lesions, comorbidities, and rotating night shift work, unless a certain covariable is the basis of the stratification.

Table S3. Subgroup analyses of the associations between daytime napping duration and overall survival among ovarian cancer patients

	Variables	Model 2 [a] HR (95% CI)	P for multiplicative interaction
**Age at diagnosis (years)**	**≤ 50**		0.23
	No (0 hours/day)	1.00 (Ref)	
	Yes (> 0 hours/day)	0.97 (0.48, 1.98)	
	the change of every 30 minutes	0.90 (0.63, 1.29)	
	**> 50**		
	No (0 hours/day)	1.00 (Ref)	
	Yes (> 0 hours/day)	**1.75 (1.06, 2.90)**	
	the change of every 30 minutes	**1.30 (1.06, 1.58)**	
**Body mass index (kg/m^2)**	**< 24**		0.12
	No (0 hours/day)	1.00 (Ref)	
	Yes (> 0 hours/day)	1.03 (0.62, 1.71)	
	the change of every 30 minutes	1.00 (0.79, 1.27)	
	**≥ 24**		
	No (0 hours/day)	1.00 (Ref)	
	Yes (> 0 hours/day)	**2.23 (1.14, 4.38)**	
	the change of every 30 minutes	**1.45 (1.08, 1.97)**	
**Menopausal status**	**No**		0.62
	No (0 hours/day)	1.00 (Ref)	
	Yes (> 0 hours/day)	1.18 (0.53, 2.62)	
	the change of every 30 minutes	1.13 (0.76, 1.69)	
	**Yes**		
	No (0 hours/day)	1.00 (Ref)	
	Yes (> 0 hours/day)	1.41 (0.88, 2.24)	
	the change of every 30 minutes	1.18 (0.96, 1.46)	
**Parity**	**≤ 1**		0.15
	No (0 hours/day)	1.00 (Ref)	
	Yes (> 0 hours/day)	1.16 (0.73, 1.85)	
	the change of every 30 minutes	1.06 (0.85, 1.33)	

	**≥ 2**		
	No (0 hours/day)	1.00 (Ref)	
	Yes (> 0 hours/day)	2.48 (0.99, 6.17)	
	the change of every 30 minutes	1.26 (0.93, 1.71)	
**Histological type**	**Serous**		0.29
	No (0 hours/day)	1.00 (Ref)	
	Yes (> 0 hours/day)	1.22 (0.77, 1.93)	
	the change of every 30 minutes	1.12 (0.90, 1.39)	
	**Non-serous**		
	No (0 hours/day)	1.00 (Ref)	
	Yes (> 0 hours/day)	1.73 (0.72, 4.17)	
	the change of every 30 minutes	1.21 (0.86, 1.70)	
**Residual lesions**	**No**		0.22
	No (0 hours/day)	1.00 (Ref)	
	Yes (> 0 hours/day)	**1.70 (1.01, 2.86)**	
	the change of every 30 minutes	1.16 (0.94, 1.42)	
	**Yes**		
	No (0 hours/day)	1.00 (Ref)	
	Yes (> 0 hours/day)	0.75 (0.39, 1.45)	
	the change of every 30 minutes	0.93 (0.66, 1.32)	

CI, confidence interval; HR, hazard ratio; Ref, reference.

HR and 95% CI were calculated with the use of the Cox proportional hazards regression model.

[a] Adjusted for age at diagnosis, body mass index, physical activity, electronic product use, smoking, alcohol drinking, tea drinking, education level, family income per month, menopausal status, parity, histological type, histopathologic grade, FIGO stage, residual lesions, comorbidities, and rotating night shift work, unless a certain covariable is the basis of the stratification.

Table S4. Subgroup analyses of the associations between sleep quality and overall survival among ovarian cancer patients

Variables		Model 2 [a] HR (95% CI)	*P* for multiplicative interaction
**Age at diagnosis (years)**	**≤ 50**		0.61
	Good (PSQI score ≤ 5)	1.00 (Ref)	
	Poor (PSQI score > 5)	**2.55 (1.25, 5.19)**	
	the change of every 1 score	1.08 (1.00, 1.16)	
	**> 50**		
	Good (PSQI score ≤ 5)	1.00 (Ref)	
	Poor (PSQI score > 5)	**2.28 (1.39, 3.73)**	
	the change of every 1 score	**1.08 (1.02, 1.14)**	
**Body mass index (kg/m^2)**	**< 24**		0.10
	Good (PSQI score ≤ 5)	1.00 (Ref)	
	Poor (PSQI score > 5)	**3.13 (1.87, 5.26)**	
	the change of every 1 score	**1.11 (1.04, 1.17)**	
	**≥ 24**		
	Good (PSQI score ≤ 5)	1.00 (Ref)	
	Poor (PSQI score > 5)	1.65 (0.88, 3.11)	
	the change of every 1 score	1.07 (0.99, 1.16)	
**Menopausal status**	**No**		0.15
	Good (PSQI score ≤ 5)	1.00 (Ref)	
	Poor (PSQI score > 5)	**4.07 (1.66, 9.97)**	
	the change of every 1 score	1.09 (1.00, 1.20)	
	**Yes**		
	Good (PSQI score ≤ 5)	1.00 (Ref)	
	Poor (PSQI score > 5)	**2.13 (1.34, 3.38)**	
	the change of every 1 score	**1.09 (1.03, 1.15)**	
**Parity**	**≤ 1**		0.26
	Good (PSQI score ≤ 5)	1.00 (Ref)	
	Poor (PSQI score > 5)	**2.82 (1.73, 4.61)**	
	the change of every 1 score	**1.09 (1.03, 1.15)**	

	**≥ 2**		
	Good (PSQI score ≤ 5)	1.00 (Ref)	
	Poor (PSQI score > 5)	1.71 (0.79, 3.66)	
	the change of every 1 score	1.07 (0.97, 1.18)	
**Histological type**	**Serous**		0.98
	Good (PSQI score ≤ 5)	1.00 (Ref)	
	Poor (PSQI score > 5)	**2.42 (1.52, 3.87)**	
	the change of every 1 score	**1.08 (1.02, 1.14)**	
	**Non-serous**		
	Good (PSQI score ≤ 5)	1.00 (Ref)	
	Poor (PSQI score > 5)	**2.83 (1.17, 6.88)**	
	the change of every 1 score	**1.13 (1.03, 1.24)**	
**Residual lesions**	**No**		0.35
	Good (PSQI score ≤ 5)	1.00 (Ref)	
	Poor (PSQI score > 5)	**2.93 (1.74, 4.91)**	
	the change of every 1 score	**1.12 (1.06, 1.18)**	
	**Yes**		
	Good (PSQI score ≤ 5)	1.00 (Ref)	
	Poor (PSQI score > 5)	**2.12 (1.08, 4.16)**	
	the change of every 1 score	1.04 (0.95, 1.14)	

CI, confidence interval; HR, hazard ratio; Ref, reference.

HR and 95% CI were calculated with the use of the Cox proportional hazards regression model.

[a] Adjusted for age at diagnosis, body mass index, physical activity, electronic product use, smoking, alcohol drinking, tea drinking, education level, family income per month, menopausal status, parity, histological type, histopathologic grade, FIGO stage, residual lesions, comorbidities, and rotating night shift work, unless a certain covariable is the basis of the stratification.

Table S5. Subgroup analyses of the associations between sleep pattern and overall survival among ovarian cancer patients

Variables		Model 2 [a] HR (95% CI)	*P* for multiplicative interaction
**Age at diagnosis (years)**	**≤ 50**		0.39
	Early bed-early rise	1.00 (Ref)	
	Early bed-late rise	0.64 (0.24, 1.70)	
	Late bed-early rise	**4.09 (1.60, 10.47)**	
	Late bed-late rise	1.92 (0.66, 5.59)	
	**> 50**		
	Early bed-early rise	1.00 (Ref)	
	Early bed-late rise	1.27 (0.67, 2.41)	
	Late bed-early rise	1.84 (0.94, 3.58)	
	Late bed-late rise	1.86 (0.88, 3.91)	
**Body mass index (kg/m^2)**	**< 24**		0.37
	Early bed-early rise	1.00 (Ref)	
	Early bed-late rise	1.16 (0.59, 2.27)	
	Late bed-early rise	**2.93 (1.46, 5.91)**	
	Late bed-late rise	1.42 (0.63, 3.22)	
	**≥ 24**		
	Early bed-early rise	1.00 (Ref)	
	Early bed-late rise	0.72 (0.29, 1.78)	
	Late bed-early rise	2.05 (0.87, 4.85)	
	Late bed-late rise	**2.90 (1.17, 7.17)**	
**Menopausal status**	**No**		0.90
	Early bed-early rise	1.00 (Ref)	
	Early bed-late rise	0.60 (0.21, 1.70)	
	Late bed-early rise	**3.79(1.15, 12.42)**	
	Late bed-late rise	1.24 (0.40, 3.82)	
	**Yes**		
	Early bed-early rise	1.00 (Ref)	
	Early bed-late rise	1.17 (0.62, 2.20)	

	Late bed-early rise	**2.37 (1.29, 4.36)**	
	Late bed-late rise	1.96 (0.94, 4.06)	
**Parity**	**≤ 1**		0.87
	Early bed-early rise	1.00 (Ref)	
	Early bed-late rise	0.83 (0.43, 1.59)	
	Late bed-early rise	**2.19 (1.19, 4.03)**	
	Late bed-late rise	**2.05 (1.05, 4.01)**	
	**≥ 2**		
	Early bed-early rise	1.00 (Ref)	
	Early bed-late rise	1.39 (0.55, 3.55)	
	Late bed-early rise	**4.17 (1.41, 12.34)**	
	Late bed-late rise	1.87 (0.40, 8.87)	
**Histological type**	**Serous**		0.30
	Early bed-early rise	1.00 (Ref)	
	Early bed-late rise	0.85 (0.45, 1.59)	
	Late bed-early rise	**2.00 (1.06, 3.76)**	
	Late bed-late rise	**2.60 (1.35, 5.00)**	
	**Non-serous**		
	Early bed-early rise	1.00 (Ref)	
	Early bed-late rise	1.06 (0.33, 3.44)	
	Late bed-early rise	1.97 (0.72, 5.42)	
	Late bed-late rise	0.84 (0.21, 3.39)	
**Residual lesions**	**No**		**0.02**
	Early bed-early rise	1.00 (Ref)	
	Early bed-late rise	0.62 (0.30, 1.29)	
	Late bed-early rise	1.85 (0.97, 3.55)	
	Late bed-late rise	1.10 (0.54, 2.26)	
	**Yes**		
	Early bed-early rise	1.00 (Ref)	
	Early bed-late rise	2.27 (0.97, 5.29)	
	Late bed-early rise	**4.33 (1.60, 11.70)**	
	Late bed-late rise	**7.91 (2.55, 24.54)**	

CI, confidence interval; HR, hazard ratio; Ref, reference.

HR and 95% CI were calculated with the use of the Cox proportional hazards regression model.

[a] Adjusted for age at diagnosis, body mass index, physical activity, electronic product use, smoking, alcohol drinking, tea drinking, education level, family income per month, menopausal status, parity, histological type, histopathologic grade, FIGO stage, residual lesions, comorbidities, and rotating night shift work, unless a certain covariable is the basis of the stratification.

Table S6. Additive model interaction effects between sleep and confounding factors on overall survival among ovarian cancer patients

	Variables	RERI (95%CI) [a]	AP (95%CI) [a]	S (95%CI) [a]
**Age at diagnosis**	Night bedtime	-1.23 (-3.45, 0.99)	-0.54 (-1.63, 0.54)	0.51 (0.17, 1.53)
	Wake-up time	0.36 (-0.42, 1.14)	0.30 (-0.32, 0.92)	-1.27 (---, ---)
	Night sleep duration	-0.40 (-1.55, 0.74)	-0.51 (-1.74, 0.72)	-1.06 (---, ---)
	Daytime napping duration	-0.50 (-1.42, 0.41)	-0.65 (-1.82, 0.52)	-0.78 (---, ---)
	Total sleep duration	-1.71 (-4.77, 1.35)	-1.52(-3.41,0.36)	0.07(0.00, 87.10)
	Sleep quality	-0.22 (-1.76, 1.32)	-0.08 (-0.63, 0.47)	0.89 (0.41, 1.91)
	sleep pattern	-0.03 (-1.11, 1.06)	-0.02 (-0.65, 0.61)	0.96 (0.23, 4.11)
**Body mass index**	Night bedtime	0.44 (-1.02, 1.90)	0.20 (-0.39, 0.78)	1.54 (0.34, 7.09)
	Wake-up time	0.28 (-0.56, 1.11)	0.23 (-0.41, 0.87)	-2.71 (---, ---)
	Night sleep duration	-0.11 (-0.91, 0.70)	-0.16 (-1.36, 1.03)	1.48 (0.04, 60.59)
	Daytime napping duration	-0.57 (-1.36, 0.22)	-0.93 (-2.41, 0.54)	-2.14 (---, ---)
	Total sleep duration	0.59 (-0.05, 1.23)	0.99 (-0.40, 2.37)	0.41 (0.19, 0.90)
	Sleep quality	-1.24 (-2.93, 0.45)	-0.53 (-1.29, 0.22)	0.52 (0.24, 1.12)
	sleep pattern	0.10 (-0.86, -1.06)	0.07 (-0.54, 0.67)	1.22 (0.16, 9.10)
**Menopausal status**	Night bedtime	-0.33 (-1.79, 1.13)	-0.26 (-1.43, 0.91)	0.46 (0.02, 9.60)
	Wake-up time	0.26 (-0.38, 0.90)	0.44 (-0.69, 1.57)	0.61 (0.22, 1.67)
	Night sleep duration	0.11 (-0.58, 0.80)	0.30 (-1.71, 2.30)	0.85 (0.35, 2.07)
	Daytime napping duration	-0.05 (-0.76, 0.67)	-0.11 (-1.80, 1.59)	1.08 (0.29, 4.13)
	Total sleep duration	-3.69 (-10.64, 3.25)	-2.96 (-5.20, -0.73)	0.06 (0.00, 12.63)
	Sleep quality	-2.09 (-4.77, 0.59)	-1.25 (-2.53, 0.04)	0.25 (0.08, 0.78)
	sleep pattern	0.05 (-0.78, 0.89)	0.07 (-0.95, 1.08)	0.76 (0.02, 29.04)
**Parity**	Night bedtime	-0.31 (-2.21, 1.58)	-0.15 (-1.14, 0.84)	0.78 (0.16, 3.84)
	Wake-up time	0.10 (-0.87, 1.06)	0.09 (-0.73, 0.90)	3.64 (0.00, ---)
	Night sleep duration	-0.49 (-1.64, 0.65)	-0.73 (-2.40, 0.93)	-1.94 (---, ---)
	Daytime napping duration	-0.57 (-1.41, 0.27)	-1.02 (-2.85, 0.81)	-3.32 (---, ---)
	Total sleep duration	-2.61 (-6.50, 1.28)	-3.04 (-6.88, 0.81)	-0.06 (---, ---)
	Sleep quality	-0.85 (-2.45, 0.76)	-0.37 (-1.13, 0.39)	0.60 (0.24, 1.49)
	sleep pattern	0.07 (-1.02, 1.16)	0.04 (-0.62, 0.71)	1.13 (0.17, 7.73)

**Histological type**	Night bedtime	-0.35 (-2.96, 2.27)	-0.10 (-0.93, 0.72)	0.87 (0.31, 2.49)
	Wake-up time	-0.29 (-1.97, 1.39)	-0.15 (-1.10, 0.80)	0.76 (0.15, 3.97)
	Night sleep duration	-0.03 (-1.22, 1.67)	-0.02 (-1.00, 0.96)	0.89 (0.01, 112.02)
	Daytime napping duration	-0.91 (-2.20, 0.39)	-0.83 (-2.39, 0.73)	0.09 (0.00, 1195.34)
	Total sleep duration	0.47 (-0.94, 1.88)	0.41 (-0.86, 1.67)	-0.49 (---, ---)
	Sleep quality	1.33 (-1.13, 3.78)	0.29 (-0.15, 0.72)	1.57 (0.68, 3.65)
	sleep pattern	0.51 (-1.44, 2.46)	0.17 (-0.41, 0.75)	1.34 (0.44, 4.12)
**Residual lesions**	Night bedtime	**3.22 (0.07, 6.38)**	**0.58 (0.30, 0.86)**	**3.43 (1.26, 9.37)**
	Wake-up time	1.85 (0.14, 3.55)	0.61 (0.31, 0.91)	10.33 (0.15, 704.39)
	Night sleep duration	-1.35 (-.3.22, 0.52)	-1.02 (-2.39, 0.35)	0.19 (0.03, 1.48)
	Daytime napping duration	0.40 (-0.67, 1.46)	0.24 (-0.34, 0.82)	2.52 (0.08, 78.34)
	Total sleep duration	-0.98 (-3.47, 1.52)	-0.66 (-2.16, 0.85)	0.33 (0.06, 1.95)
	Sleep quality	0.33 (-1.92, 2.59)	0.07 (-0.41, 0.56)	1.11 (0.56, 2.19)
	sleep pattern	2.13 (0.45, 3.80)	0.60 (0.33, 0.87)	6.16 (0.64, 58.91)

AP, the attributable proportion due to interaction; CI, confidence interval; RERI, the relative excess risk due to interaction; S, the synergy index.

[a] Adjusted for age at diagnosis, body mass index, physical activity, electronic product use, smoking, alcohol drinking, tea drinking, education level, family income per month, menopausal status, parity, histological type, histopathologic grade, FIGO stage, residual lesions, comorbidities, and rotating night shift work, unless a certain covariable is the basis of the interaction.
